# Case report of metastatic breast cancer mimicking ileal Crohn's disease

**DOI:** 10.1016/j.ijscr.2021.106408

**Published:** 2021-09-15

**Authors:** Sandeep Maharajh, Kavi Capildeo, Mickhaiel Barrow, Shariful Islam, Vijay Naraynsingh

**Affiliations:** a Medical Associates Hospital, St Joseph, Trinidad and Tobago; b Department of Clinical Medicine, The University of the West Indies, St Augustine, Trinidad and Tobago; c Department of Cellular Pathology, Port of Spain General Hospital, Trinidad and Tobago; d Department of Clinical Surgical Sciences, The University of the West Indies, St. Augustine, Trinidad and Tobago

**Keywords:** Crohn's disease, Breast Cancer, Metastasis, Bowel obstruction, Case report

## Abstract

**Introduction:**

Lobular breast cancer (LBC) has an increased risk of gastrointestinal (GI) spread compared with ductal breast carcinoma. Breast cancer commonly metastasises to bone, lung, liver, central nervous system and rarely to the gastrointestinal tract. As the prognosis for breast cancer continues to improve with modern medical practice it is important to be aware of the various clinical presentations and the appropriate management of breast cancer metastases.

**Case presentation:**

We describe a case of a 60-year-old woman who presented with symptoms of bowel obstruction 30 months after undergoing mastectomy and adjuvant chemotherapy for LBC. A Computer Tomography (CT) scan showed terminal ileal thickening suggestive of Crohn's disease but histopathology revealed metastatic lobular carcinoma. Surgical resection to relieve her small bowel obstruction confirmed LBC.

**Clinical discussion:**

This case illustrates an unusual presentation of metastatic breast cancer causing small bowel obstruction with radiological features mimicking Crohn's disease.

**Conclusion:**

Patients with breast cancer can present with intestinal obstruction due to metastatic spread to the small intestine; this may resemble Crohn's disease clinically and radiologically.

## Highlights

• Lobular breast cancer is more likely to metastasize to the gastrointestinal tract.
• Breast cancer metastases can appear radiologically as Crohn’s disease.
• Endoscopic biopsies in cases of GI spread of breast cancer can be nondiagnostic.

## 1 Introduction

Breast Cancer preferentially metastasizes to bone, lung, liver and the central nervous system [1]. The presence of these metastases is associated with poor clinical outcomes [2]. Breast cancer metastasis presenting as bowel obstruction is distinctly rare. We report a case of invasive LBC with subsequent spread to the stomach and terminal ileum who was managed at a tertiary health institution. This work has been reported in line with the SCARE Criteria, 2020 [3].

## 2 Presentation of case

A 60-year-old Caucasian female with stage T3N1M0 left LBC underwent mastectomy and axillary node dissection with prophylactic right mastectomy and adjuvant radiotherapy and chemotherapy; chemotherapy regimen included doxyrubicin, adriamycin and paclitaxel followed by anastrazole. She had no significant medical history and was not on any daily medication. She had one sister who had a history of breast cancer and her mother had ovarian cancer. She was a former tobacco smoker. Subsequent and regular PET-CT scans showed no evidence of local recurrence, up to 2 years post-operatively.

**Fig. 1:**
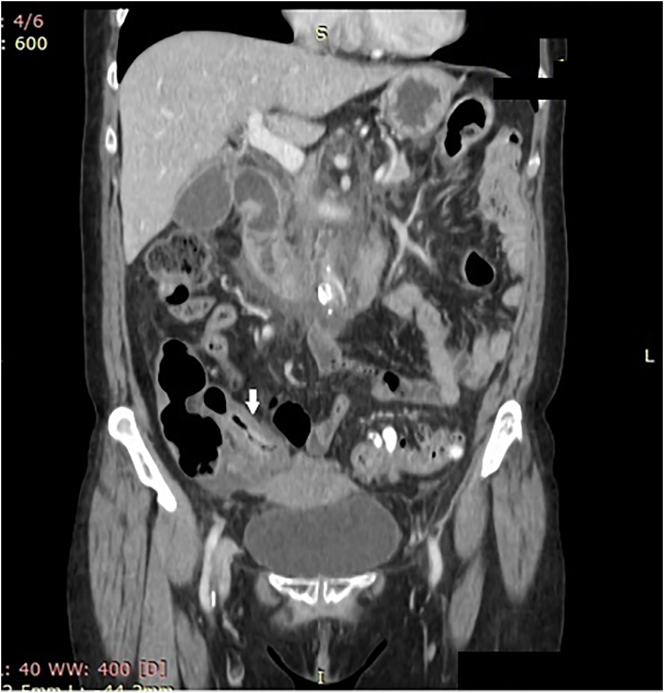
CT scan of abdomen and pelvis showing diffuse, fusiform thickening of the terminal ileum (arrowed) and enhancement of the luminal surface representing inflammation.

Thirty months after initial treatment and regular follow up, she presented to the accident and emergency department with intermittent colicky abdominal pain and distension over a period of 10 days with worsening of symptoms over the last day. There were no signs of peritonitis and she was hemodynamically stable. A CT scan of the abdomen and pelvis revealed tubular circumferential wall thickening with hyperenhancement of the terminal ileum (Fig. 1). A presumptive diagnosis of Crohn's disease was made. These features were not apparent on imaging performed 3 months prior. She was started on prednisolone and referred for inpatient colonoscopy.

Colonoscopy revealed mucosal oedema, erythema and narrowing of the terminal ileum, with abrupt change at the ileo-caecal junction. Oesophagogastro-duodenoscopy showed an abnormal area in the gastric fundus with thickening and erythema of the rugal folds. Biopsies from the gastric and ileal lesions showed metastatic LBC. The tumour was positive for Estrogen Receptor (ER), Progestogen Receptor (PR) and negative for HER2/neu. The immunoprofile was identical to the original breast cancer.

**Fig. 2:**
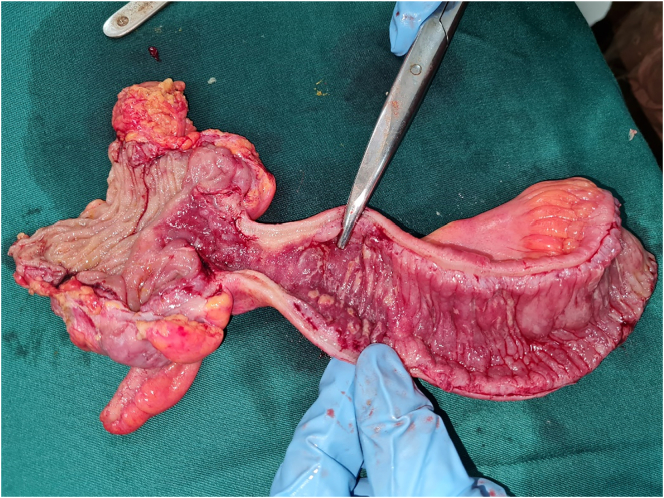
Resected gross specimen showing diffuse thickening of the terminal ileum corresponding to the radiographic finding in Fig. 1.

**Fig. 3:**
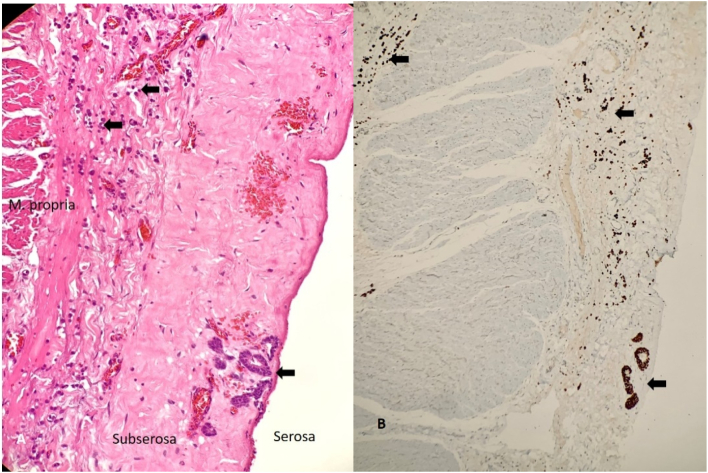
(A) Histology showing metastatic lobular breast carcinoma (arrows) involving the serosa, subserosa and muscularis propria. The mucosa and submucosa were not involved (not shown). (B) GATA-3 highlighting tumour cells (arrows). Magnification ×100.

Surgery was performed by the head surgeon (VN) to alleviate her worsening abdominal pain and sub-acute intestinal obstruction (Fig. 2). Through a midline laparotomy, the distal 25 cm of ileum was thickened and firm: up to the ileocaecal junction. The proximal unaffected bowel was mildly dilated. The distal 35 cm of ileum, caecum and proximal ascending colon were resected with an ileo-colic anastomosis. No mass lesion was visible or palpable in the stomach. Histology of the resected ileum showed metastatic LBC infiltrating from the serosa to the muscularis propria, sparing submucosa and mucosa (Fig. 3). The tumour was positive for GATA3 confirming its origin from breast. She recovered uneventfully and had complete relief of her previous severe colicky abdominal pain. She was discharged day 3 post-op for oncological treatment for her gastric metastasis. Over the following 6 months she had made progressive clinical improvement.

## 3 Discussion

The incidence of gastrointestinal spread of breast cancer has been estimated between 4%-18% of reported metastases; while rare, it is well established [4]. As the treatment for breast cancer continues to improve, this case underscores the importance of being aware of potential sites of metastatic disease encountered in modern practice.

In an extensive study of 2605 patients with breast cancer, Borst et al. showed LBC has a higher risk of spread to the GI and gynaecological tracts, the peritoneum and retroperitoneum than ductal carcinoma [5]. In this study, less than 1% of patients had spread to the GI tract. The reason for this increased risk of GI spread is uncertain but could be related to loss of the cell–cell adhesion molecule, E-cadherin, in these tumours [6]. Contrastingly, metastatic ductal carcinoma is more likely to present with hepatic, pulmonary and cerebral metastases [7].

As in this case, metastasis to the stomach is the most common GI site of spread by breast cancer, accounting for 70% of cases of GI spread [8]. In this same study of 2605 patients, 8% of cases involved the small intestine and 1% involved either the anus or oropharynx. Early diagnosis of primary & secondary GIT cancers and multidisciplinary treatment strategies will substantially increase the survival of both patients. Therefore, though metastatic breast cancer uncommonly involves the gastrointestinal tract, it must be considered in the differential if a history of breast cancer is elicited.

In this case, a diagnosis of Crohn's disease was suspected as the cause of diffuse distal ileal thickening; (Fig. 1*)*. Though quite rare, metastatic breast cancer presenting like Crohn's disease has been reported. Rubin et al. reported a case of vague GI symptoms in a known breast cancer patient. MR enterography showed thickening and hyperenhancement of distal ileum and discontinuous segments of colon with segmental stricturing. Histopathology confirmed invasive lobular carcinoma [9]. Penn et al. reported a case of altered bowel habit in a patient with breast cancer. Colonoscopy showed multiple skip lesions and rectal stricturing. Biopsy revealed invasive lobular carcinoma [10]. Nazereno et al. in reported a case of partial bowel obstruction in which enteroclysis showed thickened gastric rugae and multiple ileal strictures. Gastric biopsies showed adenocarcinoma of breast origin [11]. Accordingly, it is well documented that metastatic breast carcinoma can clinically and radiologically resemble Crohn's disease.

Another difficulty faced is that endoscopic biopsies can be negative in more than 50% of cases [12], [13]. Tumours can invade from the outer serosal aspect of the bowel and might not extend into the mucosa or submucosa- areas typically sampled during endoscopy. Further investigations, including peritoneal fluid cytology and open or laparoscopic biopsies, might be needed for definitive diagnosis.

The management of patients with metastatic disease to the GI tract can be challenging. Surgical intervention is often necessary to relieve emergent conditions such as bowel obstruction. However, in one study of 73 patients, palliative surgical intervention showed no overall survival benefit while treatment with systemic chemotherapy or tamoxifen had a positive overall survival benefit [14]. Similarly, a series examining 78 cases of gastric spread of breast cancer showed that hormonal therapy improved overall survival while surgical intervention or chemotherapy did not [15]. Furthermore, two similar cases have been identified with both metastases to the stomach and small bowel at differing times in disease progression [11]. Our case therefore highlights the uncommon scenario of simultaneous gastric and small intestinal spread of LBC.

In summary, several learning points should be noted. This case highlights a rare presentation of bowel obstruction due to metastatic lobular breast cancer. Additionally, it exemplifies a unique radiological finding of metastatic lobular breast cancer resulting in an enhancing, thickened terminal ileum on a contrast CT study that can mimic Crohn's disease.

## 4 Conclusion

If a history of breast cancer exists, particularly lobular carcinoma, the possibility of GI metastatic disease should be considered in patients complaining of gastrointestinal symptoms. Though an uncommon site of metastatic disease, breast cancer can mirror clinical and radiological features of Crohn's disease. Furthermore, endoscopic evaluation can be falsely reassuring as spread might not involve of the mucosa and submucosa of the bowel. Knowledge of these hazards as well as astute clinical acumen are of key importance in diagnosis and management.

## Consent

Written informed consent was obtained from the patient for publication of this case report and accompanying images. A copy of the written consent is available for review by the Editor-in-Chief of this journal on request.

## Sources of funding

None declared.

## Ethical approval

Ethical approval has been granted by the local ethics committee.

## Author contribution

Dr. Sandeep Maharajh-original drafting, conceptualization, data curation.

Professor Vijay Naraynsingh-performed surgery, Project administration, review and editing of paper.

Dr. Shariful Islam-Surgeon, validation.

Dr. Kavi Capildeo-Oncologist, validation.

Dr. Mickhaiel Barrow-Formal analysis of pathology specimens and review of manuscript.

Guarantor.

## Provenance and peer review

Not commissioned, externally peer-reviewed.

## Financial support

This report has received no funding.

## Declaration of competing interest

None. All authors confirm no potential or competing conflicts of interests exist.
